# Donor BMSC‐derived small extracellular vesicles relieve acute rejection post‐renal allograft through transmitting Loc108349490 to dendritic cells

**DOI:** 10.1111/acel.13461

**Published:** 2021-09-09

**Authors:** Zhi‐gang Wang, Hong‐en Xu, Fu‐min Cheng, Jie Zhang, Yong‐hua Feng, Dan‐hua Liu, Wen‐jun Shang, Gui‐wen Feng

**Affiliations:** ^1^ Department of Kidney Transplantation The First Affiliated Hospital of Zhengzhou University Zhengzhou China; ^2^ Precision Medicine Center of Zhengzhou University Academy of Medical Sciences Zhengzhou University Zhengzhou China

**Keywords:** acute rejection, dendritic cells, extracellular vesicles, Loc108349490, renal allograft

## Abstract

Bone marrow‐derived mesenchymal stem cell (BMSC)‐derived small extracellular vesicles (sEVs) are potent candidates for the suppression of acute rejection post‐renal allograft and have been reported to halt dendritic cells (DCs) maturation. However, whether BMSC‐derived sEVs mitigate acute rejection post‐renal allograft by targeting DCs is still unclear. In this study, donor BMSC‐derived sEVs (sEVs) relieved the inflammatory response and suppressed mature DCs (mDCs) location in kidney grafts, and increased regulatory T (Treg) cell population in the spleens of the rats that underwent kidney allograft. In lipopolysaccharide (LPS)‐stimulated immature DCs (imDCs), sEVs suppressed the maturation and migration of DCs and inactivated toll‐like receptor 4 (TLR4) signaling. Compared with LPS‐treated imDCs, imDCs treated with LPS+sEVs promoted CD4^+^T cells differentiated toward Treg cells. Subsequently, we found that Loc108349490, a long non‐coding RNA (lncRNA) abundant in sEVs, mediated the inhibitory effect of sEVs on DC maturation and migration by promoting TLR4 ubiquitination. In rats that underwent an allograft, Loc108349490 deficiency weakened the therapeutic effect of sEVs on acute rejection. The present study firstly found that sEVs alleviated acute rejection post‐renal allograft by transferring lncRNA to DCs and screened out the functional lncRNA loaded in sEVs was Loc108349490.

## Graphical abstract

This study reports that the administration of donor BMSC‐derived sEVs efficiently alleviates inflammatory response in kidney grafts post‐renal allograft by suppressing DC maturation and promoting the Treg cell population. The immunoregulatory effect of donor BMSC‐derived sEVs is attributed to Loc108349490, which mediated the maturation and migration of DCs by promoting TLR4 degradation.

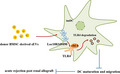

## 1 INTRODUCTION

Receiving a renal allograft is a more effective intervention than chronic dialysis for patients suffering from end‐stage renal disease, which greatly prolongs the survival of patients and improves their quality of life (Alelign et al., 2018). Unfortunately, acute rejection is a common complication post‐renal allograft that strongly threatens the graft and patient survival rates (Jehn et al., 2019). The activation of recipient T lymphocytes by donor antigens is the central event in the occurrence of acute rejection (Zhuang & Lakkis, 2015). As the most potent professional antigen‐presenting cells, dendritic cells (DCs) mediate both the direct and indirect presentation of donor antigens to recipient T lymphocytes and contribute to subsequent acute rejection (Hughes et al., 2020; Lin & Gill, 2016; Ren et al., 2016). The potential of DCs to trigger acute rejection post‐renal allograft is attributed to their maturation status. It has been reported that the density of activated donor DCs is closely associated with poor allograft survival (Batal, 2015) and that suppressing the maturation and migration of donor DCs efficiently alleviates graft immunogenicity (Kotsch, 2007). Moreover, the pre‐injection of donor renal–alloantigen–treated recipient immature DCs (imDCs) before renal allograft relieves the renal rejection response and upregulates the proportion of immunosuppressive regulatory T (Treg) cells in rat recipients (Na, 2016). Therefore, the suppression of DC maturation might be a feasible tactic to mitigate acute rejection post‐renal allograft.

Bone marrow‐derived mesenchymal stem cells (BMSCs) present their potential therapeutic effect on acute rejection post‐renal allograft due to their immunosuppressive property (Reinders et al., 2013, 2014). Small extracellular vesicles (sEVs) are small vesicles released from different cell types, such as BMSCs. Similar to BMSCs, BMSC‐derived sEVs also owned lower immunogenicity and play a beneficial role in acute renal allograft rejection. sEVs derived from recipient BMSCs prolong renal graft survival in mice recipients (Wu et al., 2017) and protect against renal ischemia‐reperfusion injury, which is a worrying clinical challenge during renal allograft (Wang et al., 2019). Furthermore, evidence shows BMSC‐derived sEVs impair antigen uptake by imDCs and impede DC maturation (Reis et al., 2018), suggesting the beneficial role of BMSC‐derived sEVs in acute rejection post‐renal allograft may attribute to its modulatory effect on DC.

sEVs execute their cellular function by delivering proteins, non‐coding RNAs (ncRNAs), lipids, etc., from donor cells to recipient cells (Kim et al., 2017). Of these, long non‐coding RNAs (lncRNAs), a type of ncRNAs more than 200 bps in length, have been regarded as crucial mediators of the biological effects of sEVs in several diseases, such as acute myocardial infarction (Huang et al., 2020), hypertrophic scar (Chen et al., 2019), and osteoporosis (Yang et al., 2019). In addition, the regulatory effect of lncRNAs loaded in sEVs on DC maturation has been confirmed by Li et al. (2019). Their research showed that vascular endothelial cell‐derived sEVs transferred lncRNA MALAT1 to imDCs; the overexpressed MALAT1 then activated the nuclear factor erythroid 2‐related factor pathway in imDCs and subsequently inhibited DC maturation. Nevertheless, whether lncRNA mediated the therapeutic effect of BMSC‐derived sEVs on acute rejection post‐renal allograft by influencing DC maturation remains unknown.

In this study, we explored whether BMSC‐derived sEVs alleviate acute rejection post‐renal allograft by modulating the status of DCs and screened out the functional lncRNA loaded in BMSC‐derived sEVs, hoping to open new perspectives for the clinical treatment of acute rejection post‐renal allograft.

## 2 MATERIALS AND METHODS

### 2.1 Animals

Beijing Charles River Experimental Animal Technology Co., Ltd. (China) provided the Wistar and Sprague‐Dawley (SD) rats (80 g or 250 g, male) used in this study. All animal experiments were performed with approval from the Ethics Committee of The First Affiliated Hospital of Zhengzhou University.

### 2.2 sEVs extraction and identification

BMSCs were isolated from SD rats (80 g, male) as previously described (Huang et al., 2020). In brief, the bone shaft was flushed out with Iscove's Modified Dulbecco's Medium (IMDM) containing 10% fetal bovine serum (FBS) and 2 mM EDTA. The cell suspension was centrifuged, suspended in IMDM medium containing 10% FBS, and seeded in cell‐culture dishes. Forty‐eight hours later, non‐adherent cells were removed and adherent cells were continuously cultured for 3–4 passages. To characterize the isolated BMSCs, MSC markers (CD44 and CD90; Tea et al., 2016) and hematopoietic markers (CD34 and CD45; Tea et al., 2016) were examined by flow cytometry (Figure S1a), and osteogenic differentiation and adipogenic differentiation were assessed by Alizarin Red S staining and Oil Red O staining, respectively (Figure S1b).

sEVs were isolated from the supernatant of SD BMSCs cultured for 48 h in exosome‐free IMDM (donor BMSC‐derived sEVs). sEVs were extracted by differential centrifugation according to the method described by Huang et al. (2020). Briefly, the culture medium was orderly centrifuged at 300 *g*, 3000 *g*, and 10,000 *g*, and 110,000 *g* to remove BMSCs, other debris, and vesicles with larger sizes. Following this, the supernatant was then centrifuged at 110,000 *g* and the cell pellets were resuspended in phosphate buffer (PBS) and centrifuged again at 110,000 *g* for 70 min. All centrifugation was performed at 4℃. The isolated sEVs were resuspended in PBS and identified by transmission electron microscope (Figure S1c) and Western blot (Figure S1d).

(Loc108349490)sEVs (sEVs that overexpressed Loc108349490) and (Si)sEVs (sEVs that silenced Loc108349490) were obtained by the following methods. First, the pcDNA 3.1 vector containing the cDNA sequences of Loc108349490 (pcDNA‐Loc108349490) and si‐RNA targeting Loc108349490 (si‐Loc108349490) were synthesized by GenePharma (China). Second, BMSCs isolated from SD rats were seeded in six‐well plates at a density of 1 × 10^6^ cells/well. When BMSCs reached 70% confluence, 5 µg pcDNA‐Loc108349490 or 30 pmol si‐Loc108349490 was transfected into cells with the assistance of Lipofectamine™ 3000 (Thermo Fisher, USA) or Lipofectamine® RNAiMAX reagent (Thermo Fisher). Six hours later, the medium was replaced with exosome‐free IMDM. Third, (Loc108349490)sEVs and (Si)sEVs were isolated from conditioned supernatants by differential centrifugation 48 h after transfection.

### 2.3 Experimental design and treatment

**FIGURE 1:**
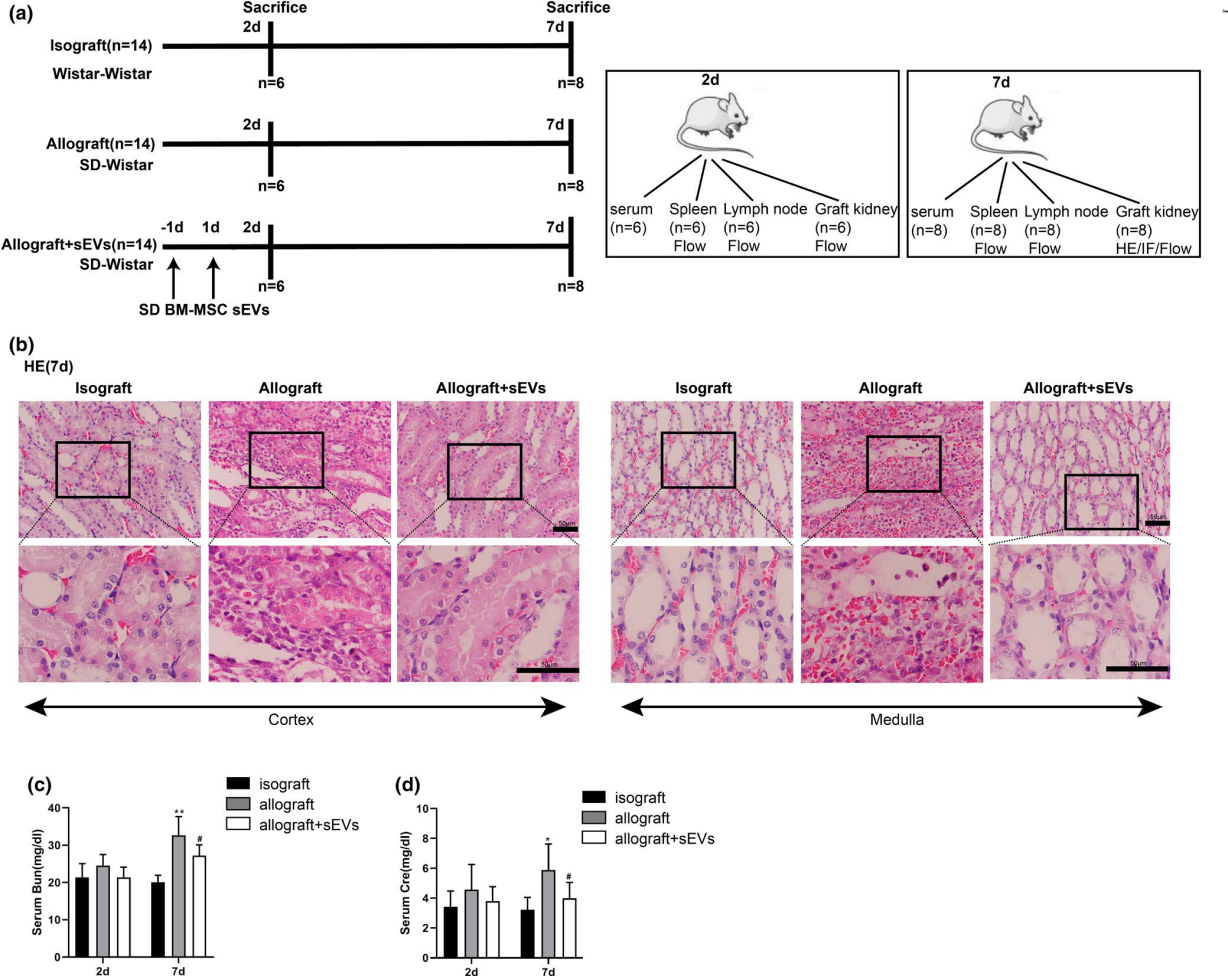
Effect of small extracellular vesicles (sEVs) derived from SD rats’ bone marrow‐derived mesenchymal stem cells (BMSCs) on inflammatory response in kidney grafts. (a) Experimental protocols of Isograft, Allograft, and Allograft+sEVs groups (*n* = 14 in each group). (b) Representative images of hematoxylin–eosin (H&E) staining (scale bar =50 µm) performed on the medulla and cortex of kidney grafts. (c) blood urea nitrogen (BUN) and serum creatinine (Cre). 2 days: *n* = 6 and 7 days: *n* = 8. *p < 0.05, **p < 0.01 vs. 7 days after Isograft. #*p* < 0.05 vs. 7 days after Allograft

For the acute rejection model, the left kidneys of SD rats (*n* = 14) were orthotopically transplanted into Wistar rats (Allograft group). The Isograft group (Wistar to Wistar, *n* = 14) served as the control group. To evaluate the effect of sEVs on acute rejection, 1.4 × 10^9^ donor BMSC‐derived sEVs were resuspended in PBS and intravenously injected into Wistar rats 1 d before and 1 d after transplantation (Allograft+sEVs group, *n* = 14). Kidney transplantation was performed as previously described (Grabner et al., 2013). In brief, the Wistar or SD rat was anesthetized, and the left kidney was perfused and removed en bloc. The donor kidney was placed in 1% heparinized saline solution at 4℃ until the Wistar recipient rat was ready to receive the transplant. The Wistar recipient rat was anesthetized and underwent a left nephrectomy. The donor kidney was placed in the posterior abdominal cavity of the recipient, and the renal arteries, renal veins, and ureters of the donor and the recipient were anastomosed using 10–0 Prolene sutures. The right kidneys of recipients were preserved, and no immunosuppressive agents were used after transplantation. Six Wistar rats from each group were sacrificed 2 days after transplantation, and the remaining Wistar rats were sacrificed 7 days after transplantation (Figure 1a). Blood, spleens, lymph nodes, and graft kidneys were collected from the rats. From the blood samples obtained from the tail vein of the rats, the levels of blood urea nitrogen (Bun) and serum creatinine (Cre) were determined utilizing commercial assay kits (Nanjing Jiancheng Bioengineering Institute, China).

### 2.4 The administration of (Si)sEVs *in vivo*

Wistar rats were assigned to Allograft, Allograft +sEVs, and Allograft + (Si)sEVs groups randomly. Eight rats were included in each group. In the Allograft group, the renal allograft was conducted as mentioned above. In the Allograft + sEVs and Allograft + (Si)sEVs groups, 1.4 × 10^9^ donor BMSC‐derived sEVs/(Si)sEVs were resuspended in PBS and intravenously injected into Wistar rats 1 day before and 1 day after the renal allograft. All rats were sacrificed 7 days after the renal allograft. Blood, spleens, lymph nodes, and graft kidneys were collected from the rats.

### 2.5 Hematoxylin–eosin (H&E) staining

Seven days after transplantation, the graft kidney was fixed, embedded, and sliced into 4 µm sections as previously described (Xu et al., 2020). Then, H&E staining was then performed on sections using the H&E Staining Kit (Beijing Solarbio Science & Technology Co., Ltd. China).

### 2.6 Immunofluorescent staining

Frozen sections of graft kidneys were thawed at room temperature and washed with PBS. After this, sections were incubated with a blocking solution and then incubated with primary antibodies overnight at 4℃. On the second day, sections were incubated with a secondary antibody and then counterstained with DAPI. An EVOS™ FL Auto 2 confocal microscope (Thermo Fisher) was used to visualize the stained sections. The primary antibodies used in this experiment were as follows: integrin αE antibody (OX62) PE (Santa Cruz Biotechnology, USA), HLA‐DRA polyclonal antibody (Proteintech, USA), and CD86 polyclonal antibody (Proteintech). The secondary antibody used in this experiment was purchased from Boster Biological Technology Co., Ltd. (China).

### 2.7 Phenotypic analysis of mDC

The single‐cell suspensions of the spleens and lymph nodes in the Allograft and Allograft + sEVs groups were prepared as previously described (Stojić‐Vukanić et al., 2015; Tentler et al., 2020) and then incubated with anti‐rat OX62 PE (eBioscience, USA), and anti‐rat major histocompatibility complex class II (MHC‐II) Alexa Fluor® 647 (Biolegend, USA) or anti‐rat CD86 FITC (Biolegend) antibodies for 30 min without light. Cells were washed three times with PBS containing 2% fetal calf serum (FCS) and 0.1% sodium azide, and the percentages of positive cells were detected by BD FACSCanto™ II Flow Cytometry System (BD Biosciences, USA) and analyzed using BD CellQuest™ Pro Software.

For kidney graft tissues, the single‐cell suspension was prepared as previously described (Koo et al., 2017) and incubated with anti‐rat DC (OX62) microbeads (Miltenyi Biotec, Germany) at 4℃ for 15 min. OX62^+^ DCs were selected using a QuadroMACS separator (Miltenyi Biotec), which was followed by incubating with anti‐rat MHC‐II Alexa Fluor® 647 and anti‐rat CD86 FITC. The proportion of MHC‐II^+^ or CD86^+^/OX62^+^ DCs was determined using the method described above.

For cell samples, the collected cells were diluted to 1 × 10^6^ cells/ml using PBS and the proportion of MHC II^+^ or CD86^+^ cells was determined by the BD FACSCantoTM II Flow Cytometry System using anti‐rat MHC‐II Alexa Fluor® 647 or anti‐rat CD86 FITC antibody (Biolegend).

The expression level of cell‐surface toll‐like receptor 4 (TLR4) on DCs was assessed by BD FACSCanto™ II Flow Cytometry System using the TLR4 polyclonal antibody (eBioscience).

### 2.8 Phenotypic analysis of Treg cells

Anti‐rat CD4 microbeads (Miltenyi Biotec) were employed for the positive selection of CD4^+^ cells from single‐cell suspensions of spleens and kidney grafts in the Isograft, Allograft, and Allograft + sEVs groups. CD4^+^T cells were then incubated with anti‐rat CD25 APC (Biolegend) and anti‐rat Foxp3 FITC antibodies (eBioscience). Multiple‐color flow cytometric analysis was conducted to determine the proportion of CD25^+^Foxp3^+^ Treg cells in CD4^+^T cells using the BD FACSCanto™ II Flow Cytometry System.

### 2.9 Generation and culture of imDCs

Immature DCs were generated from the BM cells as previously described (Lu et al., 2020). In brief, BM cells were flushed out from the femur and tibia of SD rats and cultured in RPMI 1640 medium containing 10% FCS. Three hours later, the non‐adherent cells were removed and adherent cells were continuously cultured in fresh RPMI 1640 medium supplemented with 10% FCS, 10 ng/ml recombinant rat granulocyte‐macrophage colony‐stimulating factor (GM‐CSF; PeproTech, USA), and 10 ng/ml recombinant rat interleukin‐4 (IL‐4; PeproTech) for 6 days. To assess the effect of donor BMSC‐derived sEVs on DC maturation, imDCs were divided into control, sEVs, LPS, and LPS + sEVs groups. In the control group, imDCs were normally cultured from Day 6 to Day 8. In the sEVs group, imDCs were incubated with 2.5 × 10^8^/ml sEVs for 48 h from Day 6. In the LPS group, imDCs were stimulated by 100 ng/ml bacterial lipopolysaccharide (LPS; Sigma‐Aldrich, USA) for 24 h from Day 7. In the LPS + sEVs group, 2.5 × 10^8^/ml sEVs and 100 ng/ml LPS were supplemented into the culture medium of imDCs on Day 6 and Day 7, respectively. On Day 8, cells in each group were harvested for flow cytometry, cocultured with CD4^+^T cells, or a transwell assay.

### 2.10 The coculture of imDCs and CD4^+^T cells

CD4^+^T cells were obtained from the spleens of Wistar rats using anti‐rat CD4 microbeads (Miltenyi Biotec) and cultured in anti‐CD3 (5 µg/ml; Sanquin, Netherlands) and anti‐CD28 (2 µg/ml; Sanquin)‐coated plates in RPMI 1640 medium supplemented with 10% FCS. Next, CD4^+^T cells were cocultured with control imDCs, sEVs‐treated imDCs, LPS‐treated imDCs, and LPS+sEVs‐treated imDCs for another 5 d (v/v = 1:1). The collected cells were diluted to 1×10^6^ cells/ml using PBS, and the population of Treg cells was evaluated via the method described in “Phenotypic analysis of Treg cells.”

### 2.11 sEVs labeling and tracking

Donor BMSC‐derived EVs were incubated with 1 µM PKH67 linker (Shanghai Umibio Co., Ltd., China) for 5 min and centrifuged. Next, imDCs were treated with PKH67‐labeled sEVs for an additional 24 h. The uptake of sEVs by imDCs was visualized using an EVOS™ FL Auto 2 confocal microscope (Thermo Fisher).

### 2.12 The measurement of DC migration

A transwell assay was conducted to detect the effect of donor BMSC‐derived sEVs on DC migration. LPS‐treated imDCs or LPS + sEVs‐treated imDCs (5 × 10^4^ cells/ml) were seeded into the upper chamber. The lower chamber was supplemented with 500 ng/ml recombinant rat CCL19 protein (Novus Biologicals, USA) to mimic the physiological environment of DC migration (Liu et al., 2007). Subsequently, the chamber was maintained in a 37℃ incubator with 5% CO_2_. Four hours later, cells passing through the membrane were stained with 0.1% crystal violet and photographed under a microscope.

### 2.13 Western blot

RIPA lysis buffer (Beijing Solarbio Science & Technology Co., Ltd) was employed to extract protein samples from imDCs and HEK293T cells. A BCA Protein Assay Kit (Sangon Biotech, China) was employed to determine the concentration of extracted protein samples as previously described (Roman et al., 2020). Next, 25 µg protein samples were separated by SDS‐PAGE on 12% gels and transferred to PVDF membranes. After blocking, the membranes were incubated with primary antibodies, secondary antibodies, and ECL Western blotting substrate (Beijing Solarbio Science & Technology Co., Ltd). This was followed by visualizing using a Bio‐Rad ChemiDoc XRS^+^ System. The primary antibodies used in this experiment were as follows: anti‐myeloid differentiation primary response 88 (MyD88) antibody (1:5000; Abcam, UK), anti‐TLR4 antibody (1:300; Abcam), anti‐Casitas B‐cell lymphoma‐b (cbl‐b) antibody (1:500; Santa Cruz Biotechnology), anti‐Ubiquitin antibody (1:5000; Abcam), anti‐Myc tag antibody (1:500; Abcam), anti‐HA tag antibody (1:1000; Abcam), anti‐DDDDK tag (Binds to FLAG^®^ tag sequence) antibody (1:2000; Abcam), and anti‐GAPDH antibody (1:10,000; Abcam). GAPDH was used as a loading control.

### 2.14 Quantitative real‐time PCR (qRT‐PCR)

RNA samples extracted from imDCs were reversely transcribed to cDNA using the All‐In‐One RT MasterMix (Bio Basic, Canada). Subsequently, 500 ng cDNA and Green‐2‐Go qRT‐PCR Mastermix (Bio Basic) were used to perform the qRT‐PCR analysis. The relative expression levels of TLR4, lncRNAs, CD80, and CD86 were figured out by the 2^−ΔΔCT^ method with GAPDH as an internal reference.

### 2.15 Ubiquitination assay

ImDCs were treated according to the protocol and incubated with MG132 (20 μM) 9 h before collection. Next, imDCs were lysed using NP40 lysis buffer (Boster Biological Technology Co., Ltd), and the cell lysates were then incubated with an anti‐TLR4 or anti‐Myc antibody. Twelve hours later, immunoprecipitates were obtained using protein A/G agarose beads (Thermo Fisher). The expression level of ubiquitin or HA‐ubiquitin was analyzed by Western blot.

### 2.16 RNA immunoprecipitation (RIP) assay

The RIP assay was performed to assess the interplay between Loc108349490 and TLR4/cbl‐b. Before the assay, 5 µg anti‐TLR4 or anti‐cbl‐b or anti‐rat IgG antibody was coated on 50 µl magnetic beads. ImDCs were treated with sEVs for 24 h and then treated with LPS + sEVs for another 24 h. Next, imDCs lysates were incubated overnight with a beads‐antibody complex. On the next day, the expression level of Loc108349490 in the anti‐TLR4/cbl‐b/IgG immunocomplex was analyzed by qRT‐PCR analysis.

### 2.17 Co‐IP assay

ImDCs were transfected with or without pcDNA‐Loc108349490 and then treated with LPS. Next, mDCs were lysed, and the cell lysates were incubated with anti‐TLR4/cbl‐b antibody for 12 h. On the next day, protein A/G agarose beads were employed to obtain the immunoprecipitates. The protein level of cbl‐b or TLR4 in immunoprecipitates was measured using Western blot.

HEK293T cells were transfected with Myc‐TLR4 (1 µg), Flag‐cbl‐b (0 or 1 µg), and pcDNA‐Loc108349490 (0, 1, or 4 µg). Forty‐eight hours later, HEK293T cells were lysed, and the cell lysates were incubated with an anti‐Flag/Myc antibody. The protein level of Myc‐TLR4 or Flag‐cbl‐b in immunoprecipitates was measured via the method described above.

### 2.18 Statistical analysis

GraphPad Prism 7.0 (GraphPad, USA) was used to perform statistical analyses. Data are expressed as the means ± *SD*. The statistical significance of the differences between the two experimental groups was analyzed using a Student's *t* test, and *p* < 0.05 is considered to be statistically significant.

## 3 RESULTS

### 3.1 Donor BMSC‐derived sEVs mitigated inflammatory response after renal allograft

Wistar rats were divided into Isograft, Allograft, and Allograft+sEVs groups, and the experimental protocols are presented in Figure 1a. In preliminary experiment, the survival rate of the Allograft group was lower than the Isograft group, which was then improved by the administration of donor BMSC‐derived sEVs (Figure S1e). In formal experiments, the H&E staining in Figure 1b showed a distinct histological sign of acute rejection, characterized by inflammatory cell infiltration, in the kidney grafts of the Allograft group compared with the Isograft group, whereas this sign was partly alleviated in the Allograft + sEVs group. Bun and Cre are two important indicators of renal function (Sakai et al., 2019). Herein, the levels of Bun and serum Cre were measured, and the results depicted in Figure 1c,d showed that both Bun and serum Cre were significantly increased in the Allograft group compared to the Isograft group 7 d after kidney transplantation. The administration of donor BMSC‐derived sEVs obviously decreased the Bun and serum Cre levels in the Allograft group.

### 3.2 Donor BMSC‐derived sEVs suppressed DC maturation and increased Treg cell population *in vivo*

**FIGURE 2:**
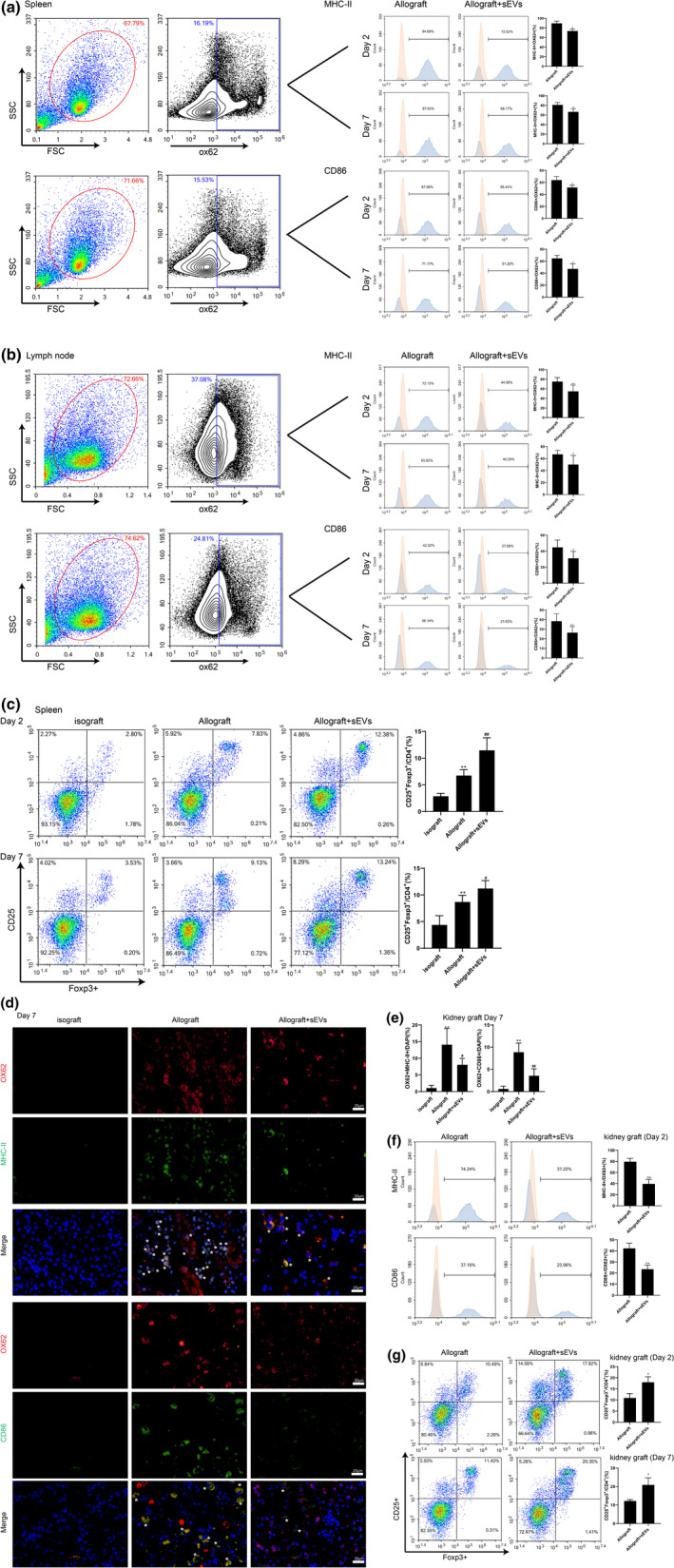
Effect of donor BMSC‐derived sEVs on dendritic cell (DC) maturation and regulatory T (Treg) cell population in vivo. The representative overlaid flow cytometry histograms indicated the expressions of major histocompatibility complex class II (MHC‐II) and CD86 in OX62^+^ gated on cells isolated from (a) spleens and (b) lymph nodes of Allograft and Allograft+sEVs rats. The bar graph showed the quantification result (*p < 0.05, **p < 0.01 vs. Allograft). (c) The proportion of splenic CD25^+^Foxp3^+^Treg/CD4^+^T cells was determined in Isograft, Allograft, and Allograft+sEVs groups 2 days and 7 days after Isograft or Allograft (**p < 0.01 vs. Isograft. #*p*<0.05, ##*p*<0.01 vs. Allograft). (d) The expressions of OX62^+^MHC‐II^+^ and OX62^+^CD86^+^ cells were detected in the kidney grafts of the Isograft, Allograft, and Allograft+sEVs groups 7 d after Isograft or Allograft by immunohistochemical staining [scale bar =25 µm; mature DCs (mDCs) were marked with white *], and the quantitation was presented in (e) (**p<0.01 vs. Isograft. #*p*<0.05, ##*p* < 0.01 vs. Allograft). Nuclei were counterstained with DAPI (blue). (f) The percentages of MHC‐II^+^/OX62^+^ and CD86^+^/OX62^+^ cells were measured in the kidney grafts of the Allograft and Allograft+sEVs groups 2 days after Allograft (**p < 0.01 vs. Allograft). (g) The proportion of CD25^+^Foxp3^+^/CD4^+^T cells was determined in the kidney grafts of the Allograft and Allograft+sEVs groups 2 and 7 days after Allograft (*p<0.05 vs. Allograft). 2 days: *n* = 6 and 7 days: *n* = 8

To evaluate the effect of donor BMSC‐derived sEVs on DC maturation, the percentage of positive cells for MHC‐II and costimulatory molecule CD86 (two cell‐surface markers of mDCs (Mellman & Steinman, 2001)) within the OX62^+^ (the specific marker of rat DC; Yang et al., 2015) DC population in the Allograft and Allograft + sEVs groups were determined. As shown in Figure 2a, the relative proportions of splenic MHC‐II^+^/OX62^+^ DCs and CD86^+^/OX62^+^ DCs of the Allograft + sEVs group were decreased compared to those of the Allograft group on Day 2 and Day 7 after the renal allograft. Similarly, the administration of donor BMSC‐derived sEVs decreased the numbers of mDCs in the lymph nodes of Allograft rats on Day 2 and Day 7 after the renal allograft (Figure 2b). Meanwhile, the ratio of splenic CD25^+^Foxp3^+^ (two surface markers of Treg; Yu et al., 2012) cells in CD4^+^T cells was upregulated in the Allograft group compared to the Isograft group, and the donor BMSC‐derived sEVs augmented this ratio in the Allograft group (Figure 2c). The results of immunofluorescent staining indicated that donor BMSC‐derived sEVs lessened mDCs (OX62^+^MHC‐II^+^ or OX62^+^CD86^+^) location in the kidney grafts after the allograft (Figure 2d,e). The downregulated proportion of MHC‐II^+^/OX62^+^ and CD86^+^/OX62^+^ DCs in the kidney grafts of the Allograft + sEVs group on Day 2 after the renal allograft also supported this finding (Figure 2f). Moreover, donor BMSC‐derived sEVs distinctly increased the percentage of CD25^+^Foxp3^+^/CD4^+^T cells in the kidney grafts on Day 2 and Day 7 after the renal allograft (Figure 2g).

### 3.3 Donor BMSC‐derived sEVs repressed maturation and migration of DCs and promoted the Treg cell differentiation *in vitro*

**FIGURE 3:**
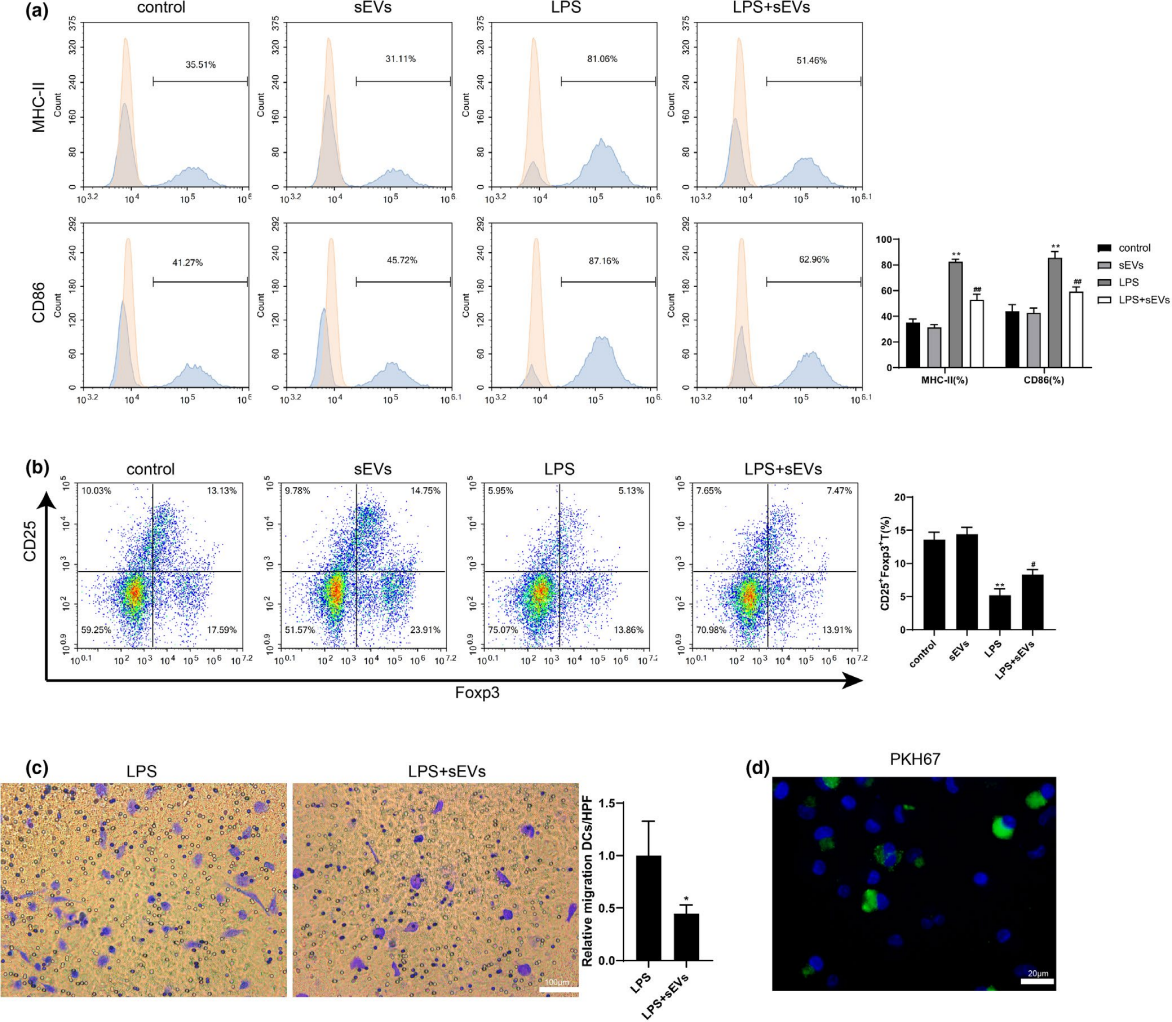
Effect of donor BMSC‐derived sEVs on maturation and migration of DC and the Treg cell differentiation *in vitro*. BM‐derived immature DCs (imDCs) were cultured under a normal condition for six days and then divided into four groups: control, sEVs, LPS, and LPS+sEVs. In the control group, imDCs were normally cultured from Day 6 to Day 8. In the sEVs group, imDCs were incubated with 2.5 × 10^8^/ml sEVs for 48 h from Day 6. In the LPS group, imDCs were stimulated by 100 ng/ml bacterial lipopolysaccharide (LPS) for 24 h from Day 7. In the LPS+sEVs group, 2.5 × 10^8^/ml sEVs and 100 ng/ml LPS were supplemented into the culture medium of imDCs on Day 6 and Day 7, respectively. (a) On Day 8, cells were harvested and the percentage of MHC‐II^+^ DCs and CD86^+^ DCs were detected (**p<0.01 vs. control. ##*p*<0.01 vs. LPS). (b) On Day 8, imDCs in each group were harvested and, respectively, cocultured with CD4^+^T cells for an additional five days. The ratio of CD25^+^Foxp3^+^ Treg cells in CD4^+^T cells was detected (**p < 0.01 vs. CD4^+^T cells cocultured with imDCs. #*p* < 0.05 vs. CD4^+^T cells cocultured with LPS‐treated imDCs). (c) Transwell assay was performed on imDCs treated with LPS or LPS+sEVs on Day 8, and quantified results were expressed as the mean numbers of cells in 10 high‐power fields (HPFs). scale bar =100 µm. *p < 0.01 vs. LPS. (d) ImDCs were incubated with donor BMSC‐derived sEVs labeled by PKH67 (green) for 24 h and then visualized by a confocal microscope (scale bar =20 µm). The nuclei were stained with DAPI. *n* = 3 in each group

Following, the influence of donor BMSC‐derived sEVs on DC maturation and function was further investigated *in vitro*. Significant upregulations in the MHC‐II^+^DCs and CD86^+^DCs population in LPS‐stimulated imDCs confirmed the maturation of imDCs under LPS stimulation (Figure 3a). In addition, the pretreatment of donor BMSC‐derived sEVs showed an ability to suppress DC maturation as evidenced by the strong reductions in MHC‐II^+^DCs and CD86^+^DCs population in LPS + sEVs‐treated imDCs (Figure 3a). Meanwhile, CD4^+^T cells cocultured with LPS‐treated imDCs presented a decreased population of CD25^+^Foxp3^+^Treg cells when compared to those cells cocultured with imDCs. Whereas the effect of LPS‐treated imDCs on Treg differentiation was partly removed by donor BMSC‐derived sEVs (Figure 3b), which suggested that donor BMSC‐derived sEVs preserved the tolerogenic ability of DCs under LPS stimulation. Furthermore, the results of the transwell assay indicated that pretreatment with donor BMSC‐derived sEVs suppressed the migration of LPS‐stimulated imDCs (Figure 3c). After that, the existence of PKH67‐labeled sEVs in imDCs confirmed that sEVs were endocytosed by imDCs and then mediated the maturation and function of DCs (Figure 3d).

### 3.4 Donor BMSC‐derived sEVs mediated the maturation and function of DCs by facilitating TLR4 ubiquitination

**FIGURE 4:**
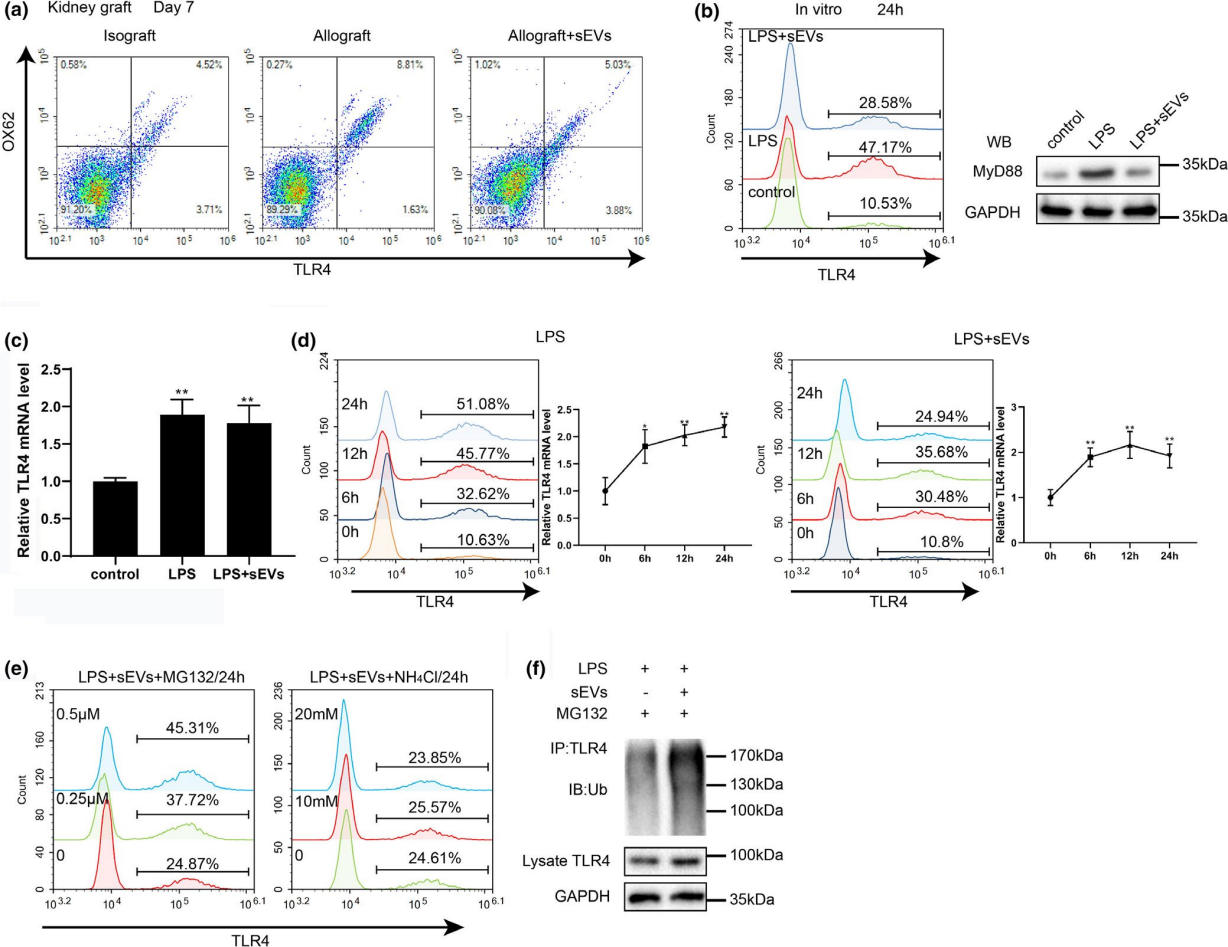
Donor BMSC‐derived sEVs facilitated ubiquitination of toll‐like receptor 4 (TLR4). (a) The percentage of TLR4^+^ OX62^+^ DCs was determined in the kidney grafts of Isograft, Allograft, and Allograft+sEVs groups 7 days after Isograft or Allograft. (b–d) ImDCs were treated with LPS or LPS+sEVs or normal medium (control) (*n* = 3 in each group). Twenty‐four hours after LPS stimulation, (b) cell‐surface TLR4 expression, myeloid differentiation primary response 88 (MyD88) protein level, and (c) TLR4 mRNA level were examined (**p < 0.01 vs. control). (d) Cell‐surface TLR4 expression and TLR4 mRNA level were measured at 0 h, 6 h, 12 h, and 24 h after LPS stimulation in imDCs treated with LPS or LPS+sEVs. *p < 0.05, **p < 0.01 vs. 0 h. (e) Cell‐surface TLR4 expression was measured in imDCs treated with LPS+sEVs+MG123 (0, 0.25, or 0.5 µM) or LPS+EVs+NH_4_Cl (0, 10, or 20 mM). (f) Ubiquitination assay of TLR4 in the lysates of imDCs treated with LPS+MG132 or LPS+sEVs+ MG132

As reported, the activation of the TLR4 signaling pathway triggers the maturation and migration of DC (Iwasaki & Medzhitov, 2004; Vargas et al., 2016; Ye et al., 2019). To determine whether TLR4 was involved in the regulatory effect of donor BMSC‐derived sEVs on DCs, the TLR4 expression level in the kidney grafts of Isograft, Allograft, and Allograft + sEVs groups was examined. As shown in Figure 4a, the population of TLR4^+^OX62^+^DCs in the kidney grafts of the Allograft + sEVs group was lessened compared with the kidney grafts of the Allograft group. Similarly, compared with LPS‐treated imDCs, the imDCs treated with LPS + sEVs presented a decreased cell‐surface level of TLR4 and a downregulated MyD88 protein level (Figure 4b), indicating that donor BMSC‐derived sEVs suppressed the activation of the TLR4 signaling pathway. Moreover, the mRNA level of TLR4, measured by qRT‐PCR, demonstrated that pretreatment with donor BMSC‐derived sEVs did not affect the TLR4 transcription (Figure 4c). We then examined the TLR4^+^ cell population and the TLR4 mRNA level in imDCs treated with LPS or LPS + sEVs 0 h, 6 h, 12 h, and 24 h after LPS stimulation. The results demonstrated that although LPS + sEVs treatment apparently lowered the cell‐surface TLR4 expression compared to LPS treatment from 12 h after treatment, there was no significant difference in the TLR4 mRNA level between the two groups (Figure 4d). These data confirmed that donor BMSC‐derived sEVs suppressed the activation of the TLR4 signaling pathway by promoting the degradation of TLR4 protein. In eukaryotic cells, most proteins are degraded through the ubiquitin–proteasome pathway or the autophagy–lysosomal pathway (Veland et al., 2019). To explore whether these pathways were involved in sEVs‐induced TLR4 degradation, imDCs were treated with LPS + sEVs + MG132 (a proteasome inhibitor) or LPS + sEVs + NH_4_Cl (a lysosome inhibitor), and the TLR4 cell‐surface expression was examined 24 h later. As shown in Figure 4e, relative to NH_4_Cl, MG132 efficiently increased the cell‐surface level of TLR4 in imDCs treated with LPS + sEVs. In addition, in the presence of MG132, sEVs reinforced the combination of TLR4 and ubiquitin in LPS‐treated imDCs (Figure 4f). This further confirmed that sEVs mediated TLR4 degradation via the ubiquitin‐proteasome pathway.

### 3.5 Loc108349490 loaded in donor BMSC‐derived sEVs mediated the regulatory effect of sEVs on DCs through TLR4

**FIGURE 5:**
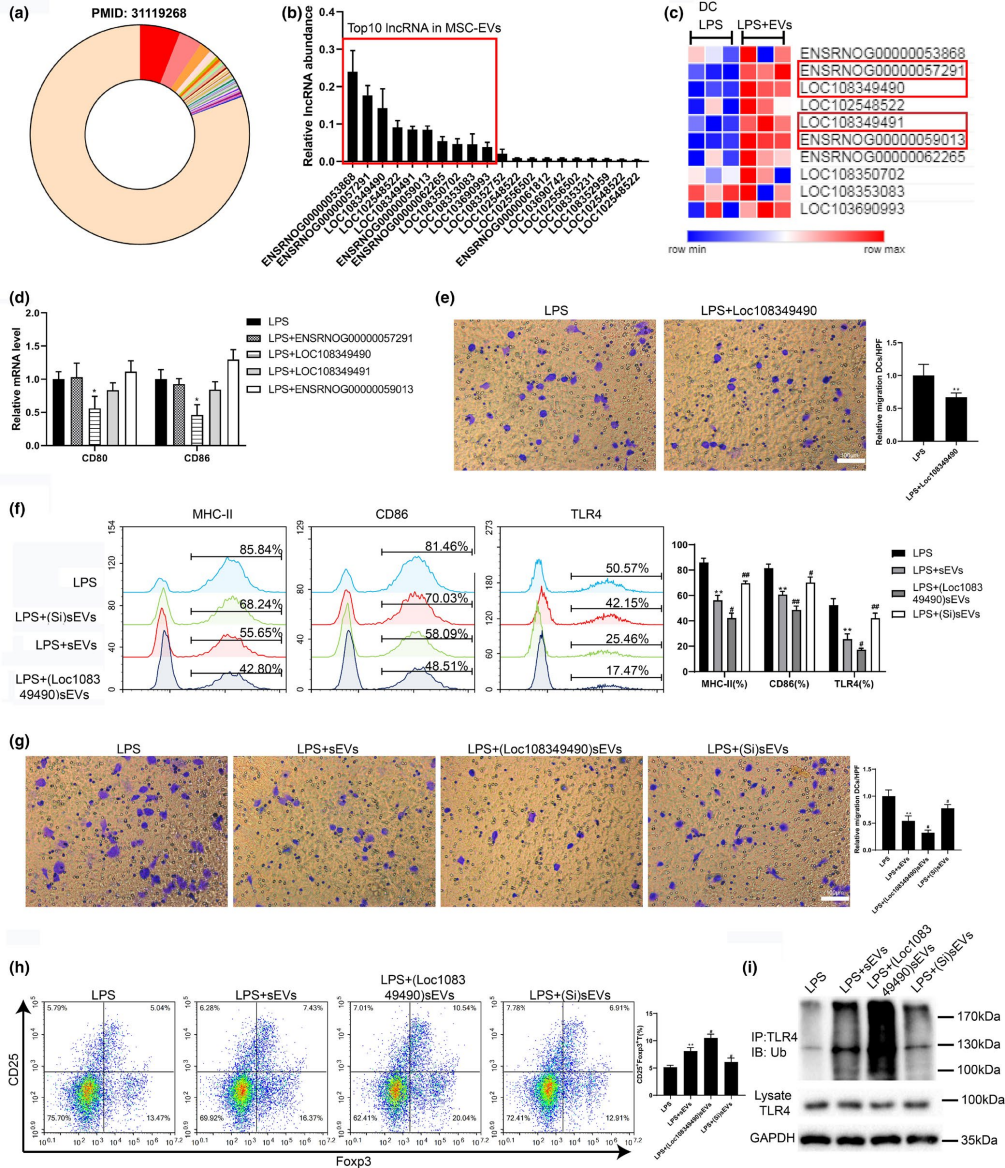
Loc108349490 loaded in donor BMSC‐derived sEVs mediated the regulatory effect of sEVs on DCs through TLR4. (a) LncRNA sequencing of SD BMSC‐derived sEVs reported by a previous study. (b) The expression levels of the top 20 lncRNA in (a) were measured in donor BMSC‐derived sEVs by qRT‐PCR. (c) The expression profile of the top 10 lncRNA in (b) was measured in imDCs treated with LPS or LPS+sEVs. (d) ImDCs were, respectively, overexpressed ENSRNOG00000057291, Loc108349490, Loc108349491, and ENSRNOG00000059013 and then stimulated by LPS for 24 h. The mRNA levels of CD80 and CD86 in imDCs were measured by qRT‐PCR. (e) Migration ability was examined by Transwell assay in imDCs treated with LPS or LPS+Loc108349490 overexpression vector (scale bar =100 µm). (f, g, and i) (Loc108349490)sEVs (sEVs that overexpressed Loc108349490) and (Si) sEVs (sEVs that silenced Loc108349490) were prepared and cocultured with imDCs 24 h before LPS stimulation. Then, (f) the percentage of MHC‐II^+^, CD86^+^, or TLR4^+^ cells, (g) migration ability (scale bar =100 µm), and (i) TLR4 ubiquitination were measured in imDCs. (h) CD4^+^T cells were, respectively, cocultured with imDCs treated with LPS, LPS+sEVs, LPS+(Loc108349490)sEVs, or LPS+(Si) sEVs for five days. The percentage of CD25^+^Foxp3^+^ Treg cells in the CD4^+^T cells was measured. *p < 0.05, **p < 0.01 vs. LPS‐treated imDCs or CD4^+^T cells cocultured with LPS‐treated imDCs. #*p* < 0.05, ##*p* < 0.01 vs. LPS+sEVs‐treated imDCs or CD4^+^T cells cocultured with LPS+sEVs‐treated imDCs. *n* = 3 in each group

Accumulating evidence confirmed that lncRNAs loaded in sEVs play a vital role in executing the cellular function of EVs (Conigliaro et al., 2015; Huang et al., 2020). To determine whether lncRNA was involved in donor BMSC‐derived sEVs‐mediated attenuation of acute rejection post‐renal allograft, the following study was conducted. Based on the lncRNA sequencing of SD BMSC‐derived sEVs conducted by Huang et al. (2020), we obtained the top 50 most abundant lncRNAs in sEVs (Figure 5a) and detected the expressions of the top 20 in donor BMSC‐derived sEVs using qRT‐PCR (Figure 5b). Next, the lncRNAs whose expression levels ranked in the top 10 in Figure 5b were selected, and their expressions were detected in imDCs treated with LPS or LPS + sEVs. As shown in Figure 5c, the expression levels of ENSRNOG00000057291, Loc108349490, LOC108349491, and ENSRNOG00000059013 in imDCs treated with LPS + sEVs were distinctly upregulated compared to those of LPS‐treated imDCs. Then, imDCs were, respectively, overexpressed ENSRNOG00000057291, Loc108349490, Loc108349491, and ENSRNOG00000059013 and then stimulated by LPS. The mRNA levels of CD80 and CD86, measured by qRT‐PCR, depicted that only Loc108349490 overexpression significantly reduced the expression levels of costimulatory molecules on DCs under LPS stimulation (Figure 5d). Meanwhile, Loc108349490 overexpression also decreased cell migration in LPS‐treated imDCs (Figure 5e). In addition, the Loc108349490 level in imDCs was not affected by the supplementation of Actinomycin D, an RNA synthesis inhibitor, indicating that the upregulation of Loc108349490 was not involved with endogenous induction (Figure S1). In all, the above data demonstrated that Loc108349490 was abundant in sEVs and might have a potential role in the maturation and migration of DCs.

To further explore whether Loc108349490 participated in the regulatory effect of sEVs on DCs, (Loc108349490)sEVs and (Si)sEVs were prepared and added into the culture medium of imDCs 24 h before LPS stimulation, and then maturation and migration of DCs were measured. The results showed that in LPS‐treated imDCs, (Loc108349490)sEVs suppressed the maturation and migration of DCs compared to EVs, whereas (Si)sEVs promoted maturation and migration of DCs (Figure 5f,g). Meanwhile, the cell‐surface TLR4 expression was decreased by (Loc108349490)sEVs and increased by (Si)sEVs in LPS‐treated imDCs (Figure 5f). Then, imDCs treated with LPS, LPS + sEVs, LPS+(Loc108349490)sEVs, and LPS+(Si)sEVs were, respectively, cocultured with CD4^+^T cells. As shown in Figure 5h, relative to CD4^+^T cells cocultured with LPS + sEVs, the proportion of CD25^+^Foxp3^+^Treg cells was upregulated in CD4^+^T cells cocultured with LPS+(Loc108349490)sEVs and downregulated in CD4^+^T cells cocultured with LPS+ (Si)sEVs. In addition, the results of the ubiquitination assay showed that TLR4 ubiquitination was reinforced by (Loc108349490)sEVs and weakened by (Si)sEVs in LPS‐treated imDCs (Figure 5i). This suggested that Loc108349490 mediated the modulatory effect of donor BMSC‐derived sEVs on the maturation and function of DCs by regulating TLR4 ubiquitination.

### 3.6 Loc108349490 reinforced cbl‐b‐mediated TLR4 ubiquitination

**FIGURE 6:**
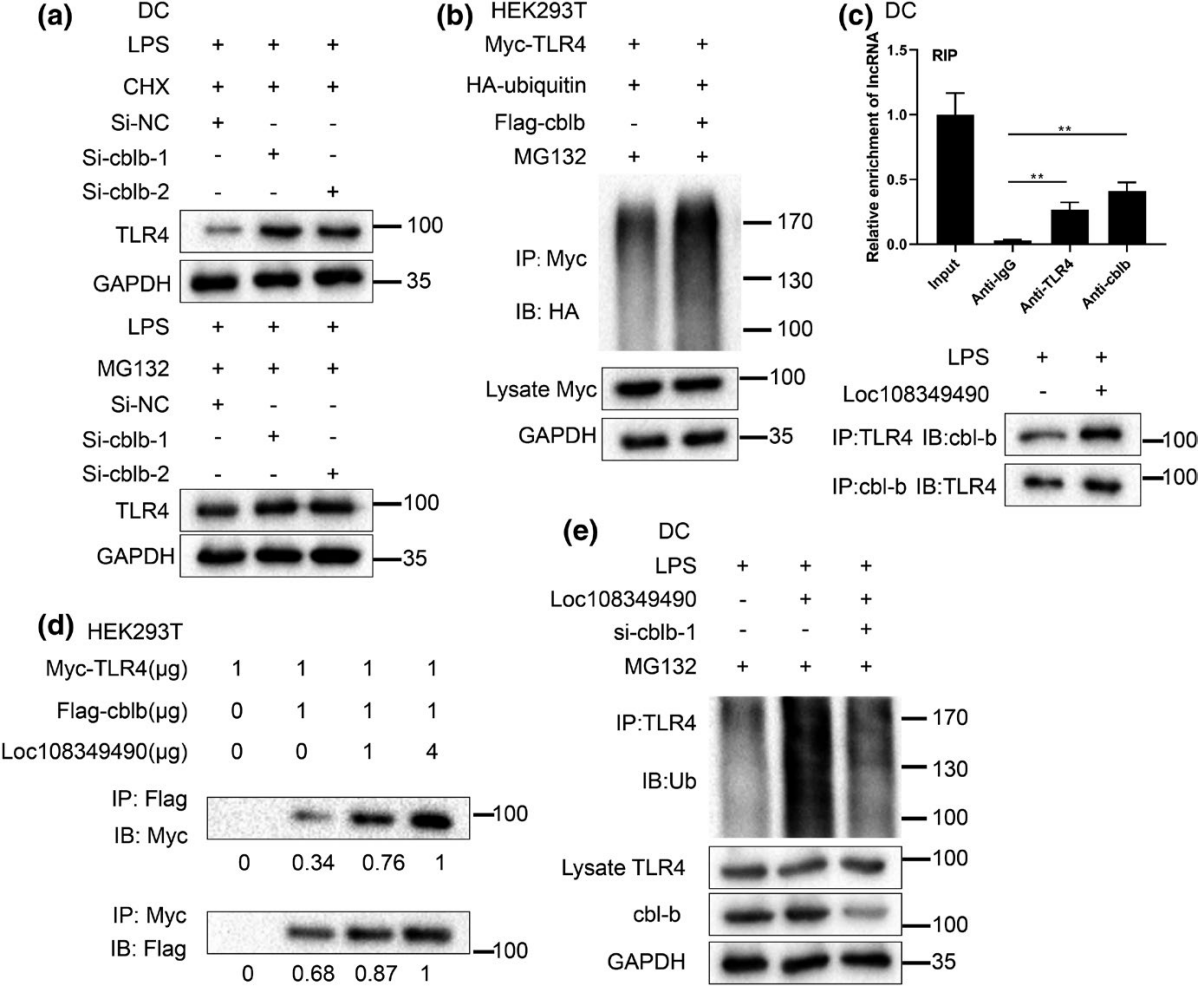
Loc108349490 reinforced cbl‐b‐mediated TLR4 ubiquitination. (a) ImDCs were transfected with si‐cbl‐b‐1, si‐cbl‐b‐2, or the negative control (si‐NC), and then treated with LPS+cycloheximide (CHX) or LPS+MG132. TLR4 protein level was measured by Western blot with GAPDH as an internal control. (b) HEK293T cells were transfected with Myc‐TLR4 and HA‐ubiquitin and/or Flag‐cbl‐b, and then treated with MG132. The ubiquitination assay of Myc‐TLR4 in the cell lysates was performed using a HA antibody. (c) Upper: RNA immunoprecipitation (RIP) followed by qRT‐PCR was performed to determine the interaction between Loc108349490 and cbl‐b/TLR4 in imDCs treated with LPS+sEVs (**p < 0.01, *n* = 3 in each group). Below: The co‐IP assay was performed to determine the combination of cbl‐b and TLR4 in imDCs treated with LPS or LPS+pcDNA‐Loc108349490. (d) HEK293T cells were cotransfected with Myc‐TLR4 (1 µg) and different doses of Flag‐cbl‐b (0 or 1 µg) and pcDNA‐Loc108349490 (0, 1, or 4 µg). The co‐IP assay was performed to determine the combination of Flag‐cbl‐b and Myc‐TLR4. (e) ImDCs were transfected with pcDNA‐Loc108349490 and/or si‐cblb‐1, and then treated with LPS and MG132. The ubiquitination assay of TLR4 in the cell lysates was performed

Cbl‐b is an E3 ubiquitin ligase responsible for the ubiquitination and degradation of TLR4 (Abe et al., 2013). In LPS‐treated imDCs, cbl‐b knockdown apparently increased the TLR4 protein level in the presence of CHX (a protein synthesis inhibitor) and failed to change the TLR4 protein level in the presence of MG132 (Fig 6a). Meanwhile, the combination of TLR4 and ubiquitin was distinctly increased by cbl‐b supplementation (Figure 6b), which confirmed that cbl‐b boosted TLR4 degradation by promoting TLR4 ubiquitination. The results of RIP showed that abundant Loc108349490 was detected in both immunoprecipitates of anti‐TLR4 antibody and anti‐cbl‐b antibody in imDCs treated with LPS + sEVs (Figure 6c), indicating that Loc108349490 was directly combined with both TLR4 and cbl‐b. Loc108349490 overexpression reinforced the interplay between TLR4 and cbl‐b (Figure 6c), and this promoting effect was stronger with the increased Loc108349490 expression level (Figure 6d). In LPS‐treated imDCs, Loc108349490 overexpression promoted the TLR4 ubiquitination, while this trend was removed by cbl‐b knockdown (Figure 6e). Taken together, these data confirmed that Loc108349490 lessened TLR4 expression by enhancing cbl‐b‐mediated TLR4 ubiquitination.

### 3.7 Donor BMSC‐derived sEVs mitigated inflammatory response post‐renal allograft through Loc108349490

**FIGURE 7:**
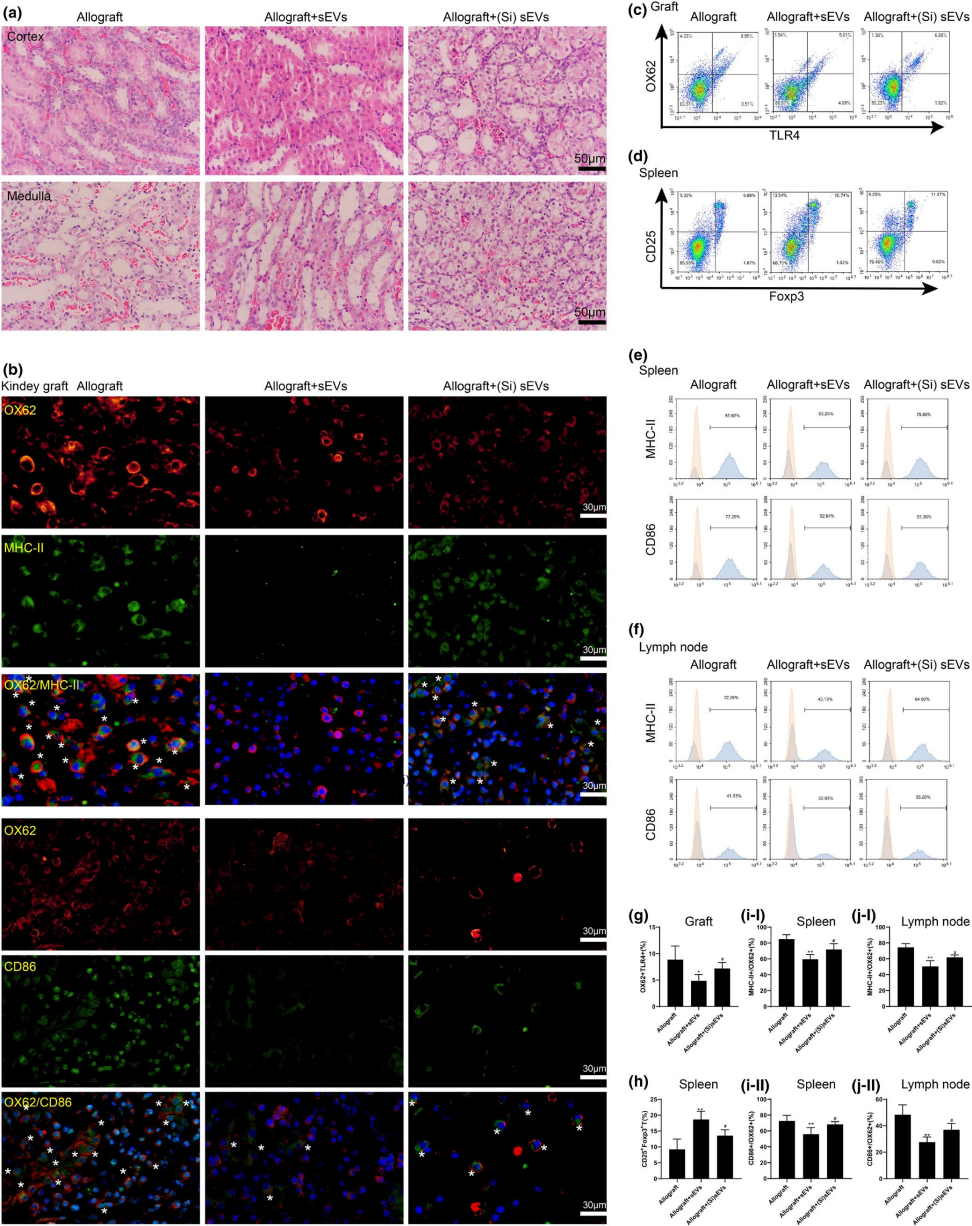
Donor BMSC‐derived sEVs relieved inflammatory response post‐renal allograft through Loc108349490. Wistar rats were divided into Allograft, Allograft+sEVs, and Allograft+(Si)sEVs groups (*n* = 8 in each group). Rats in Allograft+sEVs and Allograft+(Si)sEVs groups were received sEVs or (Si)sEVs injection 1 d before and 1 d after renal allograft, respectively. (A) Representative images of H&E staining (scale bar =50 µm). (b) The expressions of OX62^+^MHC‐II^+^ and OX62^+^CD86^+^ cells were detected in the kidney grafts of each group by immunohistochemical staining (scale bar =30 µm; mDCs were marked with white *). Nuclei were counterstained with DAPI (blue). (c) The proportion of OX62^+^TLR4^+^ cells in the kidney grafts was measured by flow cytometry, and the quantitation was presented in (g). (d) The proportion of CD25^+^Foxp3^+^ Treg cells in CD4^+^ cells in the spleen was measured by flow cytometry, and the quantitation was presented in (h). The proportions of MHCII^+^/OX62^+^ and CD86^+^/OX62^+^ DCs in the (e) spleens and (f) lymph nodes were measured by flow cytometry, and the quantitations were presented in (i) and (j), respectively. *p < 0.05, **p < 0.01 vs. Allograft. #*p* < 0.05 vs. Allograft+sEVs

To further assess whether Loc108349490 mediated the therapeutic action of donor BMSC‐derived sEVs on acute rejection post‐renal allograft, Wistar rats received sEVs or (Si)sEVs injection 1 d before and 1 d after the renal allograft, and the inflammatory response in kidney grafts was assessed. As shown in Figure 7a, compared with the Allograft + sEVs group, a more severe inflammatory response was observed in the kidney grafts of the Allograft+(Si)sEVs group. Meanwhile, the renal mDCs location was increased in Allograft+(Si)sEVs group compared to the Allograft + sEVs group (Figure 7b). Compared with sEVs, (Si)sEVs significantly upregulated the TLR4^+^OX62^+^ DCs population in the kidney graft of Allograft rats (Figure 7c,g). Furthermore, the splenic Treg cell population was downregulated (Figure 7d,h), and the mDCs population in the spleens (Figure 7e,i) and lymph nodes (Figure 7f,j) was upregulated in Allograft + (Si)sEVs group compared to the Allograft + sEVs group.

## 4 DISCUSSION

To date, the main clinical treatment for acute rejection post‐renal allograft is immunosuppressive drugs (Chadban et al., 2008). However, the chronic use of immunosuppressive therapy places patients under immunocompromised conditions and increases the risk of infection and tumors (Alberú & Urrea, 2005; Sánchez‐Fructuoso, 2008). Therefore, inducing graft immune tolerance to prevent allograft rejection is becoming a desirable goal of transplant immunologists. In the present study, we confirmed the beneficial role of donor BMSC‐derived sEVs in the induction of immune tolerance post‐renal allograft. Our data showed that the administration of donor BMSC‐derived sEVs efficiently alleviated inflammatory response in kidney grafts post‐renal allograft by suppressing DC maturation and promoting the Treg cell population. The immunoregulatory effect of donor BMSC‐derived sEVs is attributed to Loc108349490, which mediated the maturation and migration of DCs by promoting TLR4 degradation.

DCs are key participants in the occurrence of acute rejection post‐renal allograft based on their central role in the process of allorecognition through direct and indirect presentation pathways (Lin & Gill, 2016). In the direct presentation way, donor resident DCs migrate to the recipient lymphatic tissue and spleen and directly activate alloreactive T cells against donor MHC molecules (Liu et al., 2016). In the indirect presentation way, host DCs present donor‐derived antigens from donor DCs to alloreactive T cells (Que et al., 2020). These previous studies indicate that donor DC initiates acute rejection post‐renal allograft and the suppression of donor DC maturation and migration might be a feasible tactic to mitigate acute rejection post‐renal allograft. BMSC‐derived sEVs have been reported to suppress DC maturation (Reis et al., 2018) and human BMSC‐derived sEVs exhibit an immunosuppressive effect on allograft rejection post‐islet allograft in mice (Wen et al., 2016), indicating the potential role of BMSC‐derived sEVs on acute rejection post‐renal allograft by targeting DC. However, the research of Koch et al. showed that recipient BMSCs‐derived sEVs only decreased the infiltration of NK cells while not affect the renal function of kidney grafts post‐renal allograft (Koch et al., 2015). Therefore, we chose donor BMSC‐derived sEVs in this study to explore whether they could alleviate inflammatory response in kidney grafts post‐renal allograft by targeting DC.

It has been shown that DCs exhibited distinctly different biological functions in the different maturation stages; imDCs induce immune tolerance, while mDCs promote immune responses (Quah & O'Neill, 2005). The most representative characteristic of mDCs is the high expression levels of MHC II and costimulatory molecules (CD80, CD86, and CD40) on their cell surfaces (Mellman & Steinman, 2001). In the present study, the expression levels of MHC II and CD86 were significantly lessened in LPS‐induced mDCs after pretreatment with donor BMSC‐derived sEVs (Figure 3a). The ability to migrate from non‐lymphoidperipheral organs to lymph nodes is the property attained by mDCs during maturation (Britschgi et al., 2010). Using a transwell assay, we found that donor BMSC‐derived sEVs also weakened the migratory ability of imDCs under LPS stimulation (Figure 3c). These data suggested that donor BMSC‐derived sEVs inhibited the maturation of DCs. In line with our study, Reis et al. (Reis et al., 2018) reported that exposure to BMSC‐derived sEVs impaired antigen uptake by imDCs and halted DC maturation and migration. Treg cells are a subpopulation of T cells and maintain self‐immune tolerance by repressing autoreactive lymphocytes (Sakaguchi et al., 2008). The research of Kushwah et al. (2009) showed that imDCs induced tolerance in mice by inducing antigen‐specific Treg cells. The research of Xue et al. (2017) showed that single immunoglobulin IL‐1‐related receptor upregulated Treg cells in the spleens and lymph nodes by inhibiting DCs maturation, thereby inducing immune tolerance post‐islet allograft. Consistent with previous studies, increased Treg cell differentiation was observed in CD4^+^T cells cocultured with imDCs treated with LPS + sEVs relative to CD4^+^T cells cocultured with LPS‐treated imDCs (Figure 3b) in our study. In summary, the present study firstly provided evidence that donor BMSC‐derived sEVs induced immune tolerance by suppressing DC maturation and promoting imDC‐induced Treg differentiation, thereby relieving acute rejection post‐renal allograft.

TLRs are a well‐known family of pattern recognition receptors that play a vital role in the host immune system. TLR4, a member of the TLR family, is mainly expressed on DCs and contributes to the maturation of DCs and the subsequent adaptive immunity (Hu et al., 2015). Upon recognition of cognate ligands (such as LPS), TLR4 initiates a signaling cascade using both MyD88 and toll‐like receptor adaptor molecule 1 (TRIF) as signaling adaptors, leading to the activation of nuclear factor kappa‐B that can promote transcription of the costimulatory molecules on the surface of DCs (Shen et al., 2008). In the mice that underwent a renal allograft, an apparent upregulation of endogenous TLR4 ligand was observed, and the deficiencies of TLR4, MyD88, and TRIF mitigated the excretory renal allograft function and the histopathologic signs of chronic allograft damage in the chronic rejection of kidney grafts (Wang et al., 2010). In addition, Kwon et al. (2008) found that TLR4 mRNA was both increased in the renal allograft biopsies of patients with acute rejection and chronic rejection compared with that of controls. These data suggested that TLR4 signaling is the intervention target in both acute rejection and chronic rejection post‐renal allograft. In our study, the activation of TLR4 signaling was observed during the process of DC maturation (Figure 4). Donor BMSC‐derived sEVs inhibited the activation of TLR4 signaling in LPS‐treated imDCs (Figure 4) and lessened the cell‐surface level of TLR4 on OX62^+^ DCs in kidney grafts of the Allograft group (Figure 7). Compared to previous studies, the present study further clarified the vital role of TLR4 in DC maturation and acute rejection post‐renal allograft. In addition, our study clarified that TLR4 contributed to the acute rejection post‐renal allograft by mediating DC maturation and was the target molecule of donor BMSC‐derived sEVs to suppress acute rejection post‐renal allograft.

The results of the ubiquitination assay revealed that donor BMSC‐derived sEVs downregulated TLR4 expression level by promoting its ubiquitination (Figure 4). Considering that lncRNAs exhibit the ability to modulate the stability of downstream proteins and can be transferred between cells via loading in sEVs (Barile & Vassalli, 2017), we speculated that donor BMSC‐derived sEVs regulated TLR4 ubiquitination by serving as carriers of lncRNAs. Based on the lncRNA sequencing of SD BMSC‐derived sEVs conducted by Huang et al. (2020) and the results of qRT‐PCR, we determined the top 10 lncRNAs in the abundance of donor BMSC‐derived sEVs (Figure 5). Among them, the expression level of Loc108349490 in imDCs treated with LPS + sEVs was upregulated compared with that in LPS‐treated imDCs and mediated the inhibitory effect of donor BMSC‐derived sEVs on DC maturation and migration (Figure 5). In addition, consistent with our speculation, the results of RIP, co‐IP, and the ubiquitination assay confirmed that Loc108349490 reinforced TLR4 ubiquitination by enhancing the combination of TLR4 and cbl‐b, an E3 ubiquitin ligase responsible for the ubiquitination of TLR4 (Abe et al., 2013). Recently, increasing evidence has revealed the role of lncRNAs in the renal allograft. For instance Nafar et al., (2019) found the expression level of lncRNA FAS‐AS1 was higher in the peripheral blood samples of the allograft rejection group compared to the non‐rejected group in males. Pang et al. (2019) reported that lncRNA MEG3 functioned as a promoter in hypoxia‐induced kidney injury in acute renal allografts through microRNA‐181b/tumor necrosis factor α axis. However, to the best of our knowledge, the present study is the first to demonstrate the lncRNA loaded in sEVs can mitigate the acute rejection post‐renal allograft by suppressing DC maturation and migration.

In summary, our study showed that donor BMSC‐derived sEVs transferred Loc108349490 to imDCs and suppressed TLR4‐mediated DC maturation by promoting TLR4 ubiquitination, thereby inducing immune tolerance and relieving acute rejection post‐renal allograft. These results verify the beneficial role of donor BMSC‐derived sEVs in acute rejection post‐renal allograft and suggest that Loc108349490 could be an intervention target to induce graft immune tolerance.

## CONFLICT OF INTEREST

None.

## AUTHOR CONTRIBUTIONS

Zhi‐gang Wang, Wen‐jun Shang, and Gui‐wen Feng put forward the concept of the study and designed the study. Zhi‐gang Wang, Hong‐en Xu, and Fu‐min Cheng contributed to the data acquisition, prepared the manuscript, and contributed to the statistical analysis. Jie Zhang contributed to the quality control of data and algorithms. Yong‐hua Feng analyzed the data and interpretation. Dan‐hua Liu edited the manuscript. All authors read and approved the final manuscript.

## Supporting information

Fig S1

## Data Availability

Data sharing is not applicable to this article as no datasets were generated or analyzed during the current study.
