# Supplementary figures and images for: Donor BMSC‐derived small extracellular vesicles relieve acute rejection post‐renal allograft through transmitting Loc108349490 to dendritic cells
Source: Aging Cell. 2021 Sep 9;20(10):e13461. doi: 10.1111/acel.13461 (PMC8520728; doi:10.1111/acel.13461)

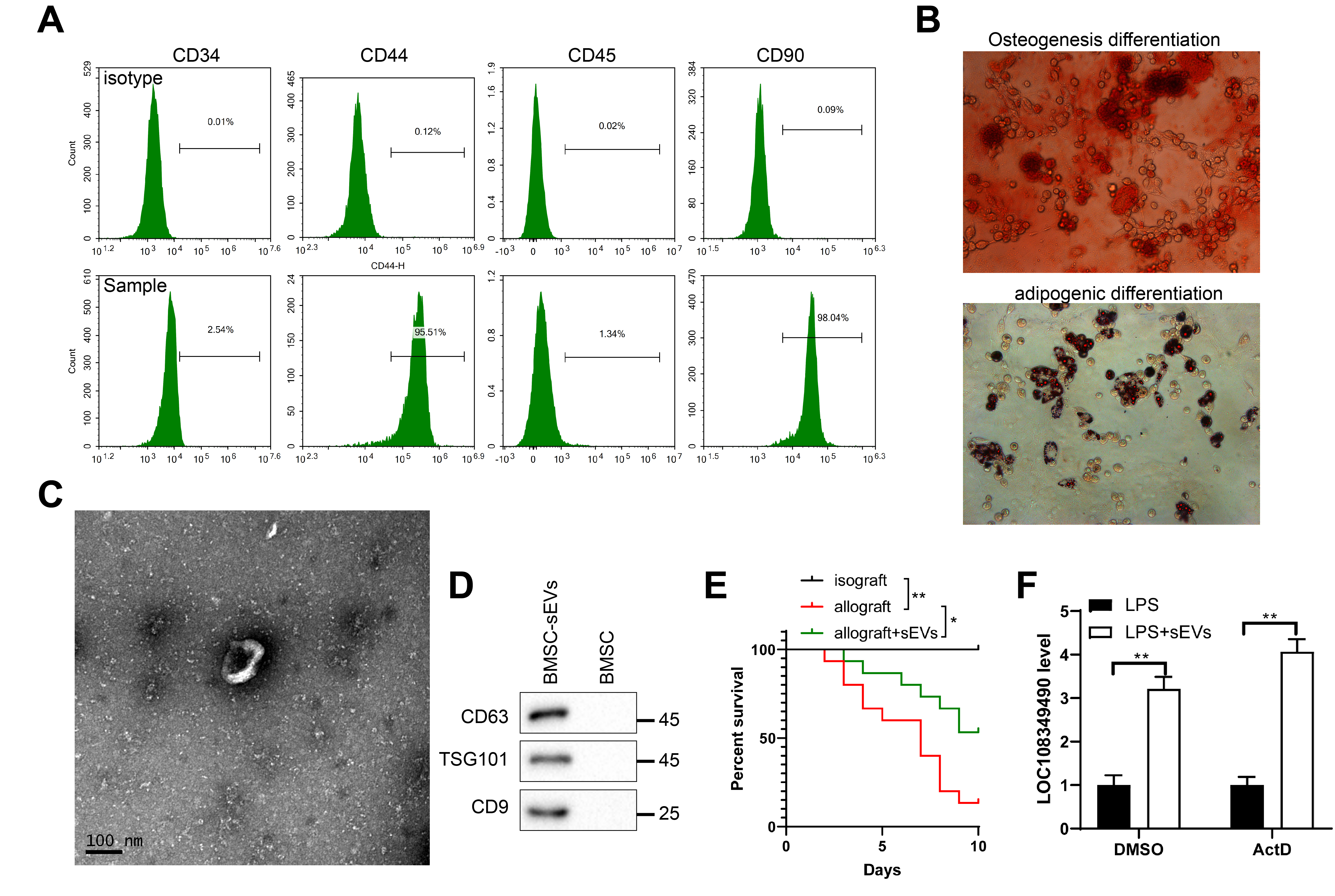

Supplement: Supplementary file 1 — Fig S1 [file ACEL-20-e13461-s001.jpg]
